# Diagnostic accuracy of synovial fluid progranulin vs. C-reactive protein in septic arthritis: a comparative biomarker study

**DOI:** 10.1186/s42358-026-00559-7

**Published:** 2026-06-27

**Authors:** Toktamış Savaş, İpek Koçer, Fatih Albayrak, Nurcihan Yavuz Savaş, Çağrı Karabulut, Bünyamin Kısacık

**Affiliations:** 1 Department of Orthopedics and Traumatology, School of Medicine, SANKO University, Gaziantep, Türkiye; 2 Department of Medical Microbiology, School of Medicine, SANKO University, Gaziantep, Türkiye; 3 Division of Rheumatology, Department of Internal Medicine, Şahinbey Research and Practice Hospital, Gaziantep University, Gaziantep, Türkiye; 4 Department of Radiology, School of Medicine, SANKO University, Gaziantep, Türkiye; 5 Department of Orthopedics and Traumatology, Pazarcık State Hospital, Pazarcık, Kahramanmaraş Türkiye; 6 Division of Rheumatology, Department of Internal Medicine, School of Medicine, SANKO University, GAZIANTEP, Türkiye

**Keywords:** C-reactive protein, Inflammatory arthritis, Progranulin, Septic arthritis, Synovial fluid

## Abstract

**Background:**

Septic arthritis (SA) is a rapidly progressive joint disease that can lead to cartilage damage if not treated promptly. A prompt and accurate distinction between SA and inflammatory arthritis (IA) is essential for establishing an optimal treatment plan. Progranulin (PRGN), an anti-inflammatory glycoprotein involved in various autoimmune diseases, has rarely been studied as a diagnostic biomarker for infectious arthritis. However, its precise role in this context remains unclear. This study aimed to evaluate the diagnostic utility of synovial fluid PRGN (SF-PRGN) in distinguishing SA from IA and osteoarthritis (OA).

**Methods:**

This single-center, cross-sectional study included 59 patients who underwent synovial fluid aspiration and were categorized into three groups: SA (*n* = 23), IA (*n* = 18), and OA (*n* = 18). SA was diagnosed based on a positive synovial fluid culture or fulfillment of clinical criteria suggestive of infection. SF-PRGN levels were measured using ELISA, and synovial fluid C-reactive protein (SF-CRP) levels were determined using an immunoturbidimetric assay.

**Results:**

Mean SF-PRGN levels were higher in the SA (339.77 ± 142.16 ng/mL) and IA (300.52 ± 159.60 ng/mL) groups than in the OA group (133.44 ± 41.77 ng/mL), indicating a statistically significant difference between the inflammatory and non-inflammatory groups (*p* < 0.05). However, the SF-PRGN did not significantly differentiate between SA and IA (*p* = 0.803). In contrast, SF-CRP levels were markedly elevated in SA (61.91 ± 46.84 mg/L) and demonstrated strong discriminatory power between SA and IA (*p* < 0.001; AUC: 0.795, *p* < 0.0001).

**Conclusion:**

Although SF-PRGN levels are elevated in inflammatory arthritis, they lack specificity for SA. SF-CRP exhibited superior diagnostic accuracy in differentiating SA from IA. These findings underscore the need for further research on reliable biomarkers of SA in larger patient cohorts.

**Clinical trial registration:**

Clinical trial number not applicable.

## Introduction

Septic arthritis (SA) is an acute joint condition that can lead to rapid joint destruction if not diagnosed and treated promptly [1, 2]. Delayed diagnosis often results in significant articular damage. However, the clinical presentation of SA may overlap with other types of arthritis, and diagnosis largely relies on culture-based methods, which are time-consuming and may delay definitive management [1]. In rheumatology practice, it is crucial to differentiate whether newly emerging arthritis is attributable to SA or inflammatory arthritis (IA), particularly in patients presenting with symptoms suggestive of diseases such as rheumatoid arthritis (RA) or psoriatic arthritis.

Progranulin (PRGN) is an 80-kDa glycoprotein originally identified as a growth factor in cancer cells and fibroblasts, with established anti-inflammatory and chondroprotective properties [3]. Its levels have been shown to increase under inflammatory conditions and correlate with disease activity in RA and systemic lupus erythematosus (SLE) [4–9]. The multifaceted inflammatory functions of PGRN in relation to TNF-α/TNFR, TLR9, IL-10, and Tregs imply that it may serve as an indicator of overall inflammation. Nevertheless, its role as a specific marker of infection in patients with septic arthritis remains to be elucidated [10]. Conventional inflammatory markers such as C-reactive protein (CRP) and erythrocyte sedimentation rate (ESR) are widely used in clinical practice; however, they often lack specificity in distinguishing SA from non-infectious arthritis, as they may be elevated in both infection and flare-ups of inflammatory diseases.

Furthermore, the limitations of culture-based diagnosis, including positive yields in only 40–54% of SA cases, delayed results (24–72 h), and low leukocyte counts in the early stages of infection [1, 11–14]—underscore the ongoing need for novel biomarkers. Recent studies have increasingly focused on synovial and serum-based biomarkers, including pentraxin-3, procalcitonin, interleukin-6, presepsin, D-lactate, and other inflammatory mediators. The aim of this focus is to improve the accuracy of early diagnosis of symptomatic arthritis and to serve as a complement to traditional culture methods [15–17]. Despite the theoretical potential of PRGN in distinguishing infectious from inflammatory processes, its diagnostic performance in differentiating SA from IA has not been thoroughly evaluated. This study aimed to address this gap in the literature by investigating the potential of PRGN as a clinical biomarker for the differential diagnosis of SA.

## Materials and methods

This cross-sectional study was conducted in a single-center setting involving both orthopedic and rheumatology outpatient clinics. Synovial fluid (SF) samples were obtained from patients presenting with arthritis of the knee, as clinically indicated by specialists in orthopedics and rheumatology.

Patients who presented with knee pain and demonstrated findings such as pain during joint movement, weight-bearing difficulty, local swelling, warmth, and tenderness on physical examination and from whom synovial fluid could be aspirated were considered eligible for inclusion. Patients were classified into the septic arthritis (SA) group if they had a positive synovial fluid culture or, in culture-negative cases, positive Gram staining or a synovial fluid leukocyte count > 50,000 cells/mm³ together with clinical findings compatible with infection, including fever, inability to bear weight, joint swelling, warmth, tenderness, and elevated inflammatory markers. Patients with rheumatologic causes of arthritis were assigned to the inflammatory arthritis (IA) group, and those with clinical and radiographic findings consistent with primary osteoarthritis were enrolled in the control group (OA).

A positive synovial fluid culture was used as the reference standard for SA diagnosis. Patients with negative cultures but with either positive gram staining or SF leukocyte counts > 50,000 cells/mm³ and clinical findings suggestive of SA were included in the SA group. The IA group included patients diagnosed with RA, PsA, and AS according to the classification criteria of the American College of Rheumatology (ACR) and European League Against Rheumatism (EULAR). Aspirations were performed in patients with SA and IA during active arthritis flares. In the OA group, aspiration was performed for therapeutic and diagnostic purposes because of symptomatic joint effusion in the affected joints. As synovial sampling from healthy individuals was not ethically permissible, patients with OA served as the noninflammatory control group.

Patients who received immunosuppressive or antibiotic therapy during arthritis flare-ups or had traumatic osteoarthritis, previous arthroplasty, or malignancy were excluded. A total of 59 patients aged 18–83 years who met the inclusion criteria were evaluated and assigned to three groups: SA (*n* = 23), IA (*n* = 18), and OA (*n* = 18) groups.

Joint aspiration was performed based on patient positioning: in the supine position, aspiration was performed from the patellofemoral joint, and in the seated position, aspiration was performed from the femorotibial compartment. Prior to aspiration, the injection site was disinfected with 10% povidone-iodine and allowed to dry for 2 min before the injection. The procedure was performed using 18G or 21G spinal needles, respectively. The aspirated synovial fluid samples were promptly transported to the microbiology laboratory for Gram staining and culture, including inoculation into BacT/ALERT FA Plus aerobic culture bottles.

SF samples were first processed in the microbiology laboratory for culture and Gram staining. Samples inoculated into BacT/ALERT FA Plus aerobic bottles (bioMérieux, France) were incubated in a BD BACTEC Fx automated system (Becton, Dickinson and Company, USA). Positive signals were subcultured on 5% sheep blood agar (RTA, Turkey) and MacConkey agar (RTA, Turkey) and evaluated after 18–24 h of incubation at 37 °C. Microorganism identification and susceptibility testing were performed using conventional methods and the BD Phoenix 100 automated system (Becton, Dickinson and Company, USA).

The SF samples remaining after microbiological evaluation were stored at -80 °C. On the day of analysis, the samples were thawed once at room temperature to minimize the preanalytical variability. CRP levels were measured using an immunoturbidimetric assay with an Alinity c analyzer (Abbott Laboratories, USA), and the same samples were used for PRGN measurements using ELISA. PRGN concentrations were determined in accordance with the manufacturer’s instructions using an ELISA kit (BT LAB, Shanghai Korain Biotech Co., Ltd., China) and were reported in ng/mL. The assay was performed using the calibration standards and standard curve provided by the manufacturer.

### Statistical analysis

The normality of numerical variables was assessed using the Kolmogorov–Smirnov test. As parametric assumptions were not met, comparisons among the groups were performed using the Kruskal–Wallis test. When a significant overall difference was detected pairwise comparisons were conducted using the Mann–Whitney U test with Bonferroni correction for multiple comparisons. Categorical variables were analyzed using the chi-square test.

Receiver Operating Characteristic (ROC) curve analysis was used to assess the discriminatory ability of the variables. Diagnostic accuracy was evaluated using ROC curves, and optimal cutoff values were determined using the Youden Index. The area under the curve (AUC) values were reported with 95% confidence intervals (CI). ROC analysis was conducted separately for SF-CRP and SF-PRGN to assess their performance in distinguishing SA from IA and OA. The resulting AUC values were used to determine the diagnostic accuracies of the biomarkers. Diagnostic performance parameters including sensitivity, specificity, positive predictive value (PPV), and negative predictive value (NPV) were calculated. A post hoc power analysis was performed to compare SF-PRGN levels between the SA and IA groups using the observed effect size, sample sizes, and an alpha level of 0.05. Statistical significance was set at *p* < 0.05. All statistical analyses were performed using IBM SPSS Statistics (version 23; IBM Corp., Armonk, NY, USA). Figures were generated using GraphPad Prism (GraphPad Software, San Diego, CA, USA) and finalized for presentation using Adobe Illustrator (Adobe Inc., San Jose, CA, USA), without altering the underlying data.

## Results

A total of 59 patients who met the inclusion criteria were enrolled and categorized into three groups: SA (*n* = 23), IA (*n* = 18), and OA (*n* = 18) groups. The mean ages were 50.7 ± 18.5 years in the SA group, 46.6 ± 11.4 years in the IA group, and 52.8 ± 18.2 years in the OA group. There were no statistically significant differences between the groups in terms of age or sex (*P* > 0.05). Demographic and clinical characteristics of the study groups are shown in Table 1.

**Table 1:** Demographic, Clinical, and Laboratory Characteristics of Patients with Septic Arthritis, Inflammatory Arthritis, and Osteoarthritis. Pairwise Comparisons of synovial fluid C-reactive protein (SF-CRP) and synovial fluid progranulin (SF-PRGN) Levels in Different Study Groups

Variables		Groups				*p*-value
		Culture (-) SA (*n* = 13) Mean ± SD Median (Min-Max)	Culture (+) SA (*n* = 10) Mean ± SD Median (Min-Max)	IA (*n* = 18) Mean ± SD Median (Min-Max)	OA (*n* = 18) Mean ± SD Median (Min-Max)	
Sex	Male	2	5	8	7	0.287^*^
	Female	11	5	10	11	
Age (years)		53.54 ± 17.75 49 (27–81)	47 ± 19.79 44.5 (21–75)	46.56 ± 11.41 43 (31–78)	52.83 ± 18.24 51.5 (14–83)	0.542^†^
SF-CRP (mg/L)		55.41 ± 34.72 64.2 (0-108.9)	70.37 ± 60.13 69.75 (0-188.1)	18.97 ± 18.17 18.25 (0-60.6)	1.53 ± 2.31 0.5 (0-8.2)	< 0.001^†^
SF-PRGN (ng/ml)		330.37 ± 156.2 294.07 (165.32-726.18)	351.99 ± 128.76 333.59 (168.75-523.79)	300.52 ± 159.6 296.39 (92.43-793.54)	133.44 ± 41.77 130.02 (52.79-211.57)	< 0.001^†^

*: The sex variable was analyzed using the Chi-square test

†: Continuous variables were analyzed using the Kruskal–Wallis test

The synovial fluid culture was positive in 10 of 23 patients (43.5%) in the SA group. The most frequently isolated microorganisms were Staphylococcus aureus (26.1%) and Streptococcus spp. (4.3%), Staphylococcus epidermidis (4.3%), Klebsiella pneumoniae (4.3%), and Group A beta-hemolytic Streptococcus (4.3%). The remaining 13 patients (56.5%) in the SA group showed culture-negative results, whereas all synovial fluid samples from the IA and OA groups were sterile.

Significant differences were observed among the groups in terms of SF-PRGN and SF-CRP levels. The SA group was further subdivided into culture-positive (SA[+]) and culture-negative (SA[–]) groups. The mean SF-PRGN levels were 330.37 ± 156.2 ng/mL in the SA(–) subgroup, 351.99 ± 128.76 ng/mL in the SA(+) subgroup, 300.52 ± 159.60 ng/mL in the IA group, and 133.44 ± 41.77 ng/mL in the OA group. The corresponding SF-CRP levels were 61.91 ± 46.84 mg/L in SA, 18.97 ± 18.17 mg/L in IA, and 1.53 ± 2.31 mg/L for OA.

**Fig. 1:**
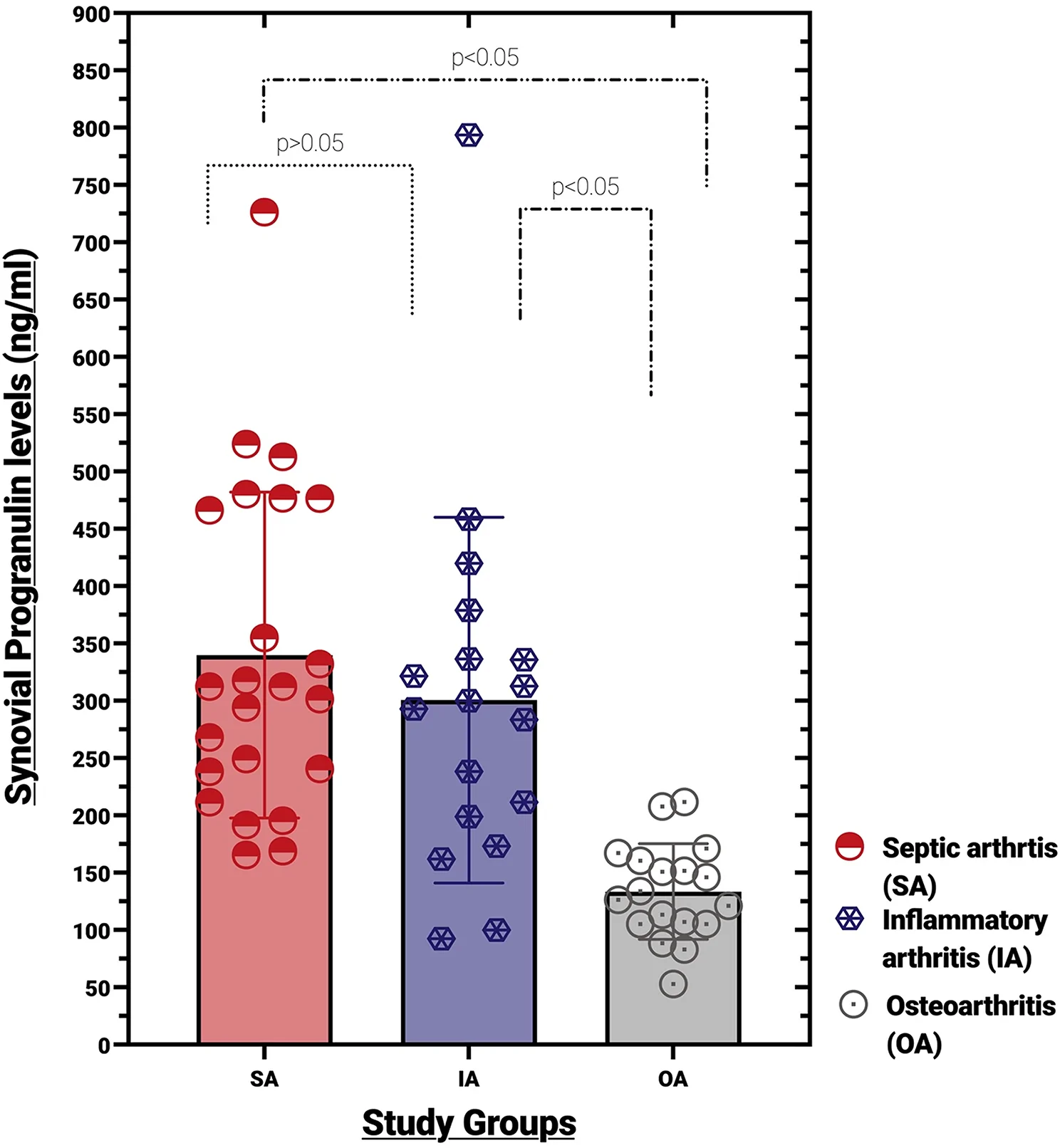
Comparison of synovial fluid progranulin (SF-PRGN) levels among patients with septic arthritis (SA), inflammatory arthritis (IA), and osteoarthritis (OA). Error bars represent standard deviation

**Fig. 2:**
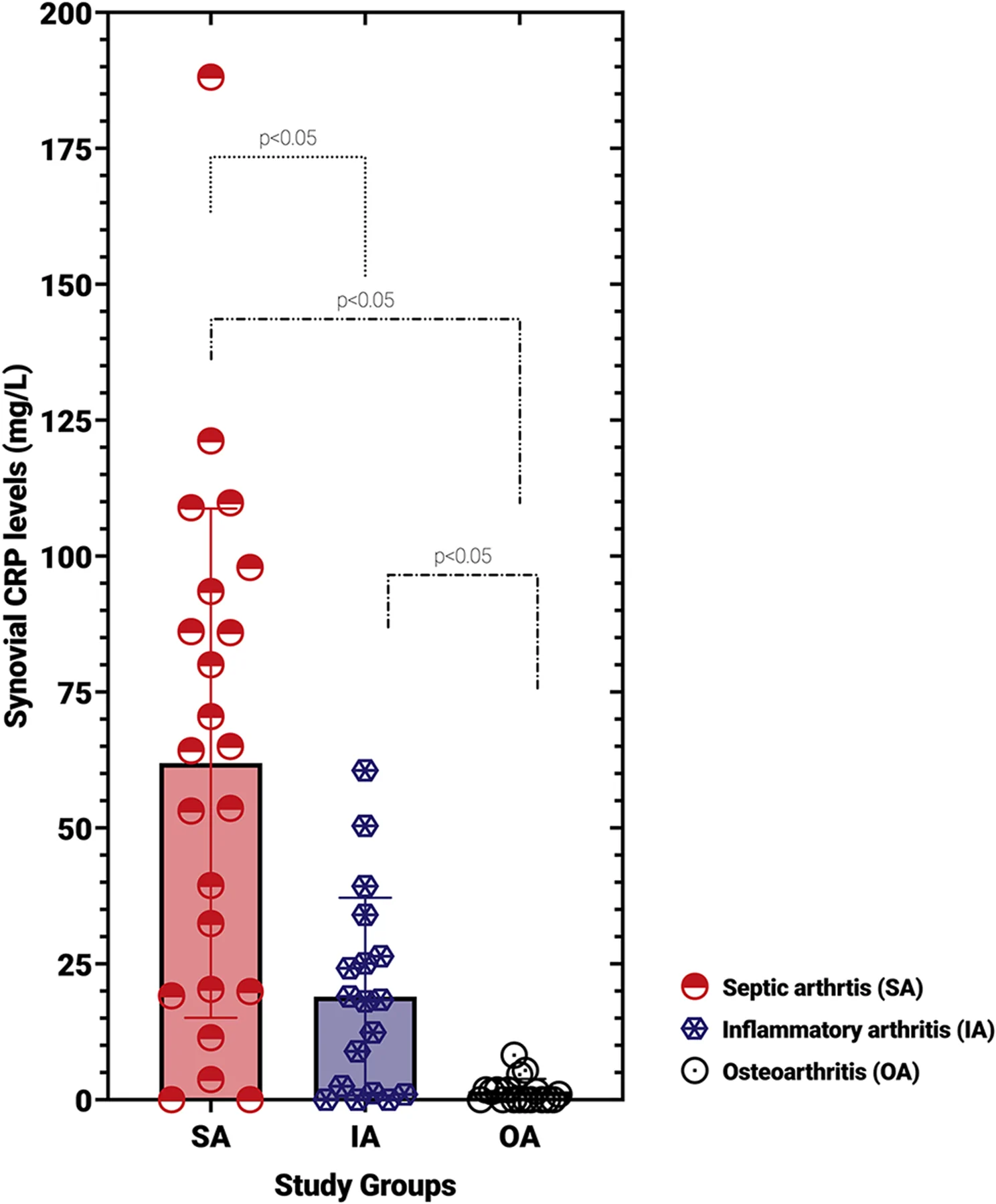
Comparison of synovial fluid C-reactive protein (SF-CRP) levels among patients with septic arthritis (SA), inflammatory arthritis (IA), and osteoarthritis (OA). Error bars represent standard deviation

The Kruskal–Wallis test was used for group comparisons of continuous variables, followed by post hoc analyses for variables that showed significant differences. The results of the post hoc comparisons are presented in Table 2, and the graphical distributions of the SF-PRGN and SF-CRP levels across all groups are shown in Figs. 1 and 2, respectively. SF-CRP levels were significantly higher in the SA group than in the IA and OA groups (*p* < 0.001 for both). No statistically significant difference was observed in SF-PRGN levels between the SA and IA groups after Bonferroni correction. For this comparison, the observed effect size was small (Cohen’s d = 0.26), and the post-hoc power analysis showed an achieved power of 12.81%. However, SF-PRGN levels were significantly higher in both SA and IA groups compared with the OA group. Subgroup analysis revealed no significant differences in SF-CRP and SF-PRGN levels between culture-positive and culture-negative SA patients (*p* = 0.983 and *p* = 0.999, respectively).

**Table 2:** Pairwise comparisons of SF-CRP and SF-PRGN levels among study groups using the Mann–Whitney U test with Bonferroni correction

Groups Compared	SF-CRP (*p*)	SF-PRGN (*p*)
SA vs. IA	< 0.011^**^	1.000
IA vs. OA	0.009^**^	< 0.001 ^**^
SA vs. OA	< 0.001^**^	< 0.001^**^
SA(-) vs. SA(+)	1.000	1.000
SA(-) vs. IA	0.024^**^	1.000
SA(-) vs. OA	< 0.001^**^	< 0.001^**^
SA(+) vs. IA	0.192	1.000
SA(+) vs. OA	0.002^**^	< 0.001^**^

**: *p* < 0.05 after Bonferroni correction

**Fig. 3:**
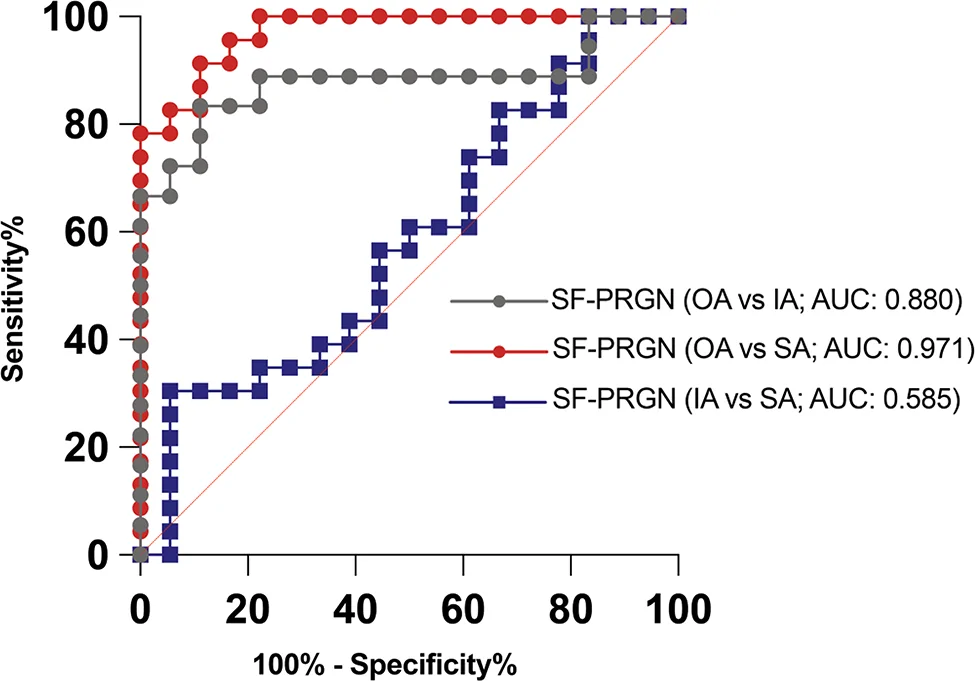
ROC curves of synovial fluid progranulin (SF-PRGN) for distinguishing septic arthritis (SA), inflammatory arthritis (IA) and osteoarthritis (OA)

**Fig. 4:**
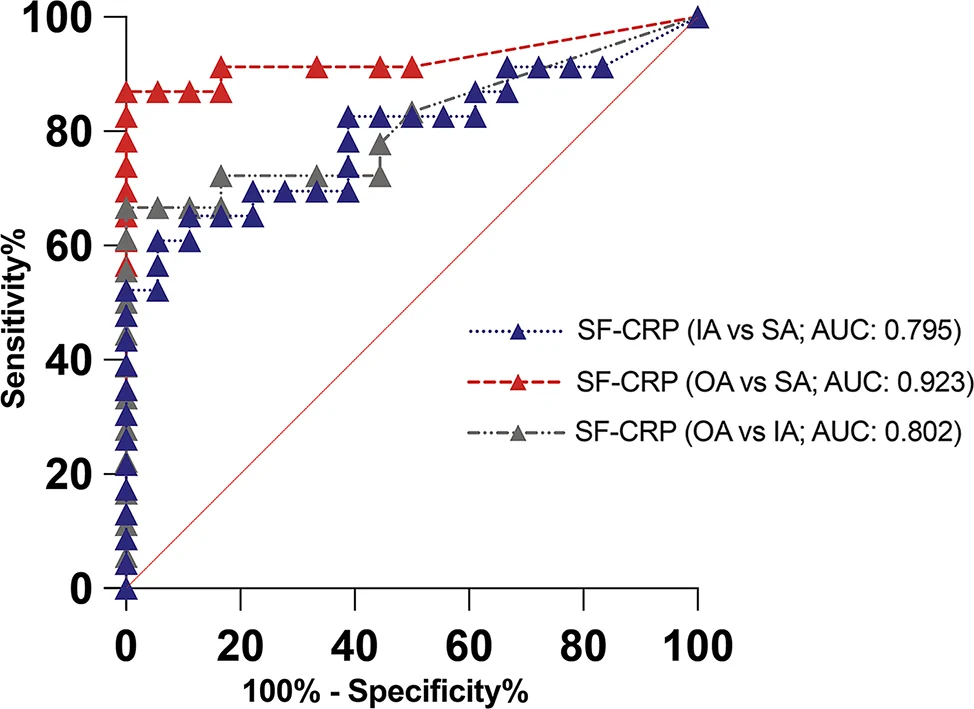
ROC curves of synovial fluid C-reactive protein (SF-CRP) for distinguishing septic arthritis (SA), inflammatory arthritis (IA) and osteoarthritis (OA)

The diagnostic performance analyses of SF-CRP and SF-PRGN are presented in Table 3 and Figs. 3 and 4. The diagnostic accuracy of SF-CRP for differentiating SA from IA was high, with an AUC of 0.795 (95% CI: 0.640–0.905, *p* < 0.0001). The optimal cut-off value was > 50.4 mg/L, with a sensitivity of 60.87% and a specificity of 94.44%. For SF-PRGN, ROC analysis yielded an AUC of 0.585 for distinguishing SA from IA, which was not statistically significant (*p* = 0.3603). Using a cutoff value of > 458.46 ng/mL, the sensitivity and specificity were 30.43% and 94.44%, respectively. To differentiate SA from OA, the AUC values were 0.923 (95% CI: 0.795–0.983, *p* < 0.0001) and 0.971 (95% CI: 0.864–0.999, *p* < 0.0001) for the SF-PRGN.

**Table 3:** Diagnostic performance of synovial fluid C-reactive protein (SF-CRP) and synovial fluid progranulin (SF-PRGN) in Differentiating septic arthritis (SA), inflammatory arthritis (IA), and osteoarthritis (OA): ROC Curve Analysis

Test	AUC (95%CI)	Cut-off Value	Sensitivity (%)	Specificity (%)
SF-CRP (SA vs. IA)	0.795 (0.640–0.905)	> 50.4 mg/L	60.87	94.44
SF-PRGN (SA vs. IA)	0.585 (0.420–0.760)	> 458.46 ng/mL	30.43	94.44
SF-CRP (SA vs. OA)	0.923 (0.795–0.983)	> 8.2 mg/L	86.96	100.00
SF-PRGN (SA vs. OA)	0.971 (0.864–0.999)	> 171.07 ng/mL	91.30	88.89
SF-CRP (IA vs. OA)	0.802 (0.636–0.916)	> 8.2 mg/L	66.67	100.00
SF-PRGN (IA vs. OA)	0.880 (0.728–0.964)	> 171.07 ng/mL	83.33	88.89

## Discussion

This study compared the diagnostic performances of synovial fluid CRP and PRGN levels in patients with SA, IA, and OA, focusing on their ability to distinguish between SA and IA. Our findings revealed that while SF-PRGN levels were significantly elevated in both the SA and IA groups compared with those in the OA group, they did not differ significantly between the SA and IA groups. In contrast, SF-CRP levels demonstrated high diagnostic accuracy in differentiating SA from IA, with significantly higher values observed in the SA group.

PRGN is a glycoprotein with anti-inflammatory and chondroprotective properties, and its expression has been shown to increase in various inflammatory arthropathies, typically linked to heightened immune activation and TNF-α mediated pathways [4–9, 18]. Based on this mechanism, a significant difference in SF-PRGN levels between patients with SA and IA was expected. Although SF-PRGN levels were higher in patients with SA than in those with IA, the difference was not statistically significant. This finding may indicate that PRGN is not an infection-specific biomarker but rather reflects broader inflammatory and tissue-repair responses. Therefore, overlapping inflammatory pathways between infectious and non-infectious arthritis may explain the limited discriminatory value of SF-PRGN in distinguishing SA from IA. Several factors may explain this outcome. First, chronic inflammatory arthritis such as RA and psoriatic arthritis, is characterized by persistent synovial proliferation and an elevated inflammatory burden. These conditions have the potential to result in the activation or suppression of various cytokine pathways, with some of these pathways having the capacity to modulate PRGN expression. In a review by Wei et al. comparing OA and IA, it was reported that PRGN expression is elevated in both diseases, although the extent of this increase varied between the two conditions [18]. This discrepancy has been attributed to the variability in the cytokine pathways involved in each disease. A similar finding was reported in a systematic review by González-Rodríguez et al., who stated that PRGN plays a role in inflammatory and degenerative musculoskeletal diseases, such as rheumatoid arthritis, systemic lupus erythematosus, and intervertebral disc degeneration, through TNF-α/TNFR signalling, TLR9 activation, IL-10 induction, and Treg-associated immune regulation [10]. Consequently, the elevated SF-PRGN levels observed in the SA and IA groups may indicate general synovial inflammatory activity rather than infection-specific inflammation. Second, although none of the patients with IA were under active immunosuppressive treatment during sample collection, some may have previously received such therapy. Given the long half-lifes and lasting biological effects of these agents, their residual influence on synovial cytokine responses and PRGN levels is plausible. However, current evidence regarding the role of PRGN in septic arthritis remains limited. While several studies have evaluated PRGN in the context of IA and OA [4, 5, 9], to our knowledge, this is one of the first studies to assess SF-PRGN levels specifically in patients with SA, providing a novel contribution to the literature. These findings may serve as a foundation for future studies on PRGN as a potential biomarker.

In our study, the proportion of culture-negative SA cases was 56.5%, corresponding to a culture positivity rate of 43.5%. Although this rate is consistent with previous reports, the relatively low culture-positive rate may be related to factors such as prior antibiotic exposure before aspiration, low bacterial burden, timing of sample collection, and limitations in the sensitivity of conventional culture methods [1, 11–14, 19]. This high proportion of culture-negative cases also represents a limitation in diagnostic accuracy and may have introduced a potential misclassification bias. The elevated SF-PRGN levels observed in both culture-positive and culture-negative SA patients suggest that PRGN may be upregulated by the inflammatory cytokine response triggered by the infectious process of SA rather than directly by bacterial proliferation. This observation supports the hypothesis that PRGN reflects the overall inflammatory response specific to SA, independent of culture positivity.

Although various studies have investigated SF-CRP, few have assessed its diagnostic value in distinguishing between SA, IA, and OA. Zamani et al. found significantly higher SF-CRP levels in patients with IA than in OA [20]. Using a methodology similar to that of our study, Streit et al. reported the highest SF-CRP levels in SA, followed by IA and OA, which is consistent with our findings [21]. Rowe et al. also demonstrated elevated SF-CRP levels in patients with IA compared to those with OA [22].

Collectively, these findings support the utility of SF-CRP as a diagnostic marker for differentiating SA from IA and OA. In the present study, the SF-CRP cutoff value of > 50.4 mg/L for differentiating SA from IA showed high specificity but moderate sensitivity, suggesting that an elevated SF-CRP level may support the diagnosis of SA, whereas lower values should not be used alone to exclude infection. On the other hand, SF-PRGN levels were higher in the inflammatory groups than in the non-inflammatory OA group but showed lower diagnostic performance than SF-CRP in distinguishing SA from IA. This may indicate that SF-CRP more closely reflects infection-associated intra-articular inflammation, whereas SF-PRGN may increase in response to both infectious and non-infectious inflammatory stimuli. However, because serum CRP was not directly evaluated in the present analysis, we cannot conclude that SF-CRP is superior to serum CRP; this issue should be investigated in future studies.

This study had some limitations. First, the relatively small sample size and single-center design may have limited the statistical power and generalizability of our findings. In particular, the post hoc power analysis showed limited statistical power for detecting differences in SF-PRGN levels between SA and IA; therefore, the non-significant result for this comparison should be interpreted with caution. The low sensitivity of SF-PRGN in differentiating SA from IA may also limit its usefulness as a standalone biomarker in urgent clinical settings. Second, although patients with osteoarthritis were used as a non-inflammatory control group because synovial fluid sampling from healthy individuals was not ethically permissible, the absence of a healthy control group may limit the interpretation of baseline SF-PRGN and SF-CRP levels in this study. Third, the inclusion of culture-negative SA cases based on clinical findings and synovial fluid leukocyte criteria may have introduced a potential misclassification bias. Therefore, the diagnostic performances of SF-PRGN and SF-CRP should be interpreted cautiously, particularly in culture-negative SA cases. Fourth, although none of the patients with IA were receiving active immunosuppressive therapy during sample collection, previous exposure to immunosuppressive agents may have influenced synovial cytokine responses and SF-PRGN levels. Fifth, the assessment was restricted to only two biomarkers, CRP and PRGN, which may not fully capture the complexity of the inflammatory process. Finally, the lack of longitudinal synovial fluid sampling limited our ability to evaluate the dynamic changes in biomarker levels throughout the disease course.

In conclusion, our results suggest that SF-CRP demonstrates high diagnostic accuracy in distinguishing SA from IA and OA and may serve as a reliable marker of infection-associated inflammation. Although SF-PRGN levels were elevated in SA and demonstrated a high AUC (0.971) in distinguishing SA from OA, its performance in differentiating SA from IA was limited by its low sensitivity (30.43%) and a lack of statistical significance. These findings suggest that PRGN may reflect overall inflammatory activity rather than act as a specific marker of infection.

Future studies with larger cohorts and prospective designs are warranted to elucidate the role of PRGN in differentiating SA from IA, particularly with careful control of clinical variables, such as disease activity, treatment history, and timing of sample collection.

## Data Availability

The datasets generated and/or analyzed during the current study are available from the corresponding author on reasonable request.
